# Midlife contributors to socioeconomic differences in frailty during later life: a prospective cohort study

**DOI:** 10.1016/S2468-2667(18)30079-3

**Published:** 2018-06-14

**Authors:** Eric J Brunner, Martin J Shipley, Sara Ahmadi-Abhari, Carlos Valencia Hernandez, Jessica G Abell, Archana Singh-Manoux, Ichiro Kawachi, Mika Kivimaki

**Affiliations:** a Research Department of Epidemiology and Public Health, University College London, London, UK; b INSERM, Centre for Research in Epidemiology & Public Health, Hôpital Paul Brousse, Bâtiment, France; c Department of Social and Behavioral Sciences, Harvard School of Public Health, Boston, MA, USA; d Clinicum, Faculty of Medicine, University of Helsinki, Finland

## Abstract

**Background:**

Health inequalities persist into old age. We aimed to investigate risk factors for socioeconomic differences in frailty that could potentially be modified through policy measures.

**Methods:**

In this multi-wave longitudinal cohort study (Whitehall II study), we assessed participants' socioeconomic status, behavioural and biomedical risk factors, and disease status at age 45–55 years, and frailty (defined according to the Fried phenotype) at baseline and at one or more of three clinic visits about 18 years later (mean age 69 years [SD 5·9]). We used logistic mixed models to examine the associations between socioeconomic status and risk factors at age 50 years and subsequent prevalence of frailty (adjusted for sex, ethnic origin, and age), with sensitivity analyses and multiple imputation for missing data.

**Findings:**

Between Sept 9, 2007, and Dec 8, 2016, 6233 middle-aged adults were measured for frailty. Frailty was present in 562 (3%) of 16 164 person-observations, and varied by socioeconomic status: 145 (2%) person-observations had high socioeconomic status, 241 (4%) had intermediate status, and 176 (7%) had low socioeconomic status, adjusting for sex and age. Risk factors for frailty included cardiovascular disease, depression, smoking, high or abstinent alcohol consumption, low fruit and vegetable consumption, physical inactivity, poor lung function, hypertension, and overweight or obesity. Cardiometabolic markers for future frailty were high ratio of total to high-density lipoprotein cholesterol, and raised interleukin-6 and C-reactive protein concentrations. The five most important factors contributing to the frailty gradient, assessed by percent attenuation of the association between socioeconomic status and frailty, were physical activity (13%), interleukin-6 (13%), body-mass index category (11%), C-reactive protein (11%), and poor lung function (10%). Overall, socioeconomic differences in frailty were reduced by 40% in the maximally-adjusted model compared with the minimally-adjusted model.

**Interpretation:**

Behavioural and cardiometabolic risk factors in midlife account for more than a third of socioeconomic differences in frailty. Our findings suggest that interventions targeting physical activity, obesity, smoking, and low-grade inflammation in middle age might reduce socioeconomic differences in later-life frailty.

**Funding:**

British Heart Foundation and British Medical Research Council.

## Introduction

Frailty is a non-specific state of disorder in an individual's physiological systems that manifests as fatigue, weakness, loss of balance, and mobility and cognitive impairment.^1^, ^2^, ^3^ It is associated with older age, chronic disease, functional impairment, and future disability, and is the most common condition leading to death in community-dwelling individuals.^4^ Frailty is the product of lifelong accumulation of biological damage and dysfunction, and its occurrence at the population level is greatest in individuals with low socioeconomic status and lowest in those with least socioeconomic disadvantage.^5^

To date, social inequalities in frailty have been documented with measures of wealth and neighbourhood deprivation and indices of relative deprivation including level of education over the life course, by use of a multidimensional frailty index.^5^, ^6^ Studies that made use of a range of socioeconomic indices in relation to the frailty phenotype,^1^ first developed in the Cardiovascular Health Study, also show gradients in frailty.^7^, ^8^, ^9^, ^10^ This inverse socioeconomic gradient is highly relevant to public health policy.^11^ However, there is little evidence on specific risk factors that might be targets for intervention to reduce socioeconomic differences in frailty.^12^

The inverse socioeconomic gradient in frailty in many countries is scientifically important in the current context of rapid global ageing.^11^ Socioeconomic gradients in health are preventable but persistent.^12^ With respect to frailty inequality, population ageing is likely to generate a large increase in poor functional health and dependency in populations of older people with socioeconomic disadvantage and thus those with fewer resources, unless prevention measures are successful.^13^, ^14^ Current healthy ageing policy places emphasis on midlife approaches to prevention.^11^, ^15^, ^16^ However, the evidence for efficacy of such measures is incomplete. Previous studies examined risk factors measured at age 60 years or older, when intervention might be too late.^8^, ^9^, ^17^ This study addresses this gap in the evidence by analysing the role in frailty inequality of behavioural and biomedical risk factors and disease status at age 50 years. Our aim was to examine the contribution of modifiable behavioural and biomedical risk factors and disease status at age 50 years to the occurrence of later life frailty and its socioeconomic gradient.

**Research in context**

**Evidence before this study**

We searched PubMed for epidemiological studies of frailty with no restriction on study location, with the search terms “ageing”, “frailty”, “prospective”, and “socioeconomic” between June 28, and Sept 30, 2017, with no language restrictions. Additionally, we hand searched reference lists in relevant papers. Social inequalities in development of frailty in old age have been documented, using a range of socioeconomic indices, in many countries. However, evidence is scarce on the role of specific risk factors in midlife that might be targets for intervention to reduce the socioeconomic gradient in frailty.

**Added value of this study**

We examined a broad range of risk factors at age 50 years that might account for the socioeconomic differences in frailty (Fried phenotype) at median age 18 years later in the Whitehall II cohort study, according to Civil Service employment grade. The five most important contributing factors were low physical activity, poor lung function, high body-mass index, high serum interleukin-6, and high C-reactive protein. The examined risk factors together accounted for 40% of the socioeconomic gradient in frailty.

**Implications of all the available evidence**

In light of rapid ageing of the global population, the observed inverse socioeconomic gradient in frailty is important, and relevant to public health policy. Interventions targeting physical inactivity, obesity, smoking, and low-grade inflammation in midlife might reduce socioeconomic differences in later-life frailty. Replication of our findings elsewhere is needed. The degree to which such policies would reduce frailty inequality depends on the assumption that the risks are reversible and that there is societal commitment to change the socioeconomic distribution of risk factors across the population.

## Methods

### Study design and participants

In this prospective, cohort study, we analysed data from the Whitehall II study, a longitudinal cohort study of British civil servants.^18^ The study began in 1985 in participants aged 35–55 years, with repeated data collection every 2–3 years. Participants had a clinical screening of behavioural and biomedical factors in 1985–88, 1991–94, 1997–99, 2002–04, 2007–09, 2012–13, and 2015–16 and additionally completed a questionnaire on behavioural risk factors in 1989–90, 1995–96, 2001, and 2006. The component measures of Fried's frailty phenotype were measured at the fifth, sixth, and seventh clinic visits.^1^ The study was approved by the University College London Medical School Committee on the ethics of human research. All participants provided informed written consent.

### Frailty

Frailty was defined according to the Fried method.^1^ The following five components were measured: walking speed,^16^ grip strength,^19^ weight loss, self-reported exhaustion, and energy expenditure from self-reported physical activity. Cutoff points for poor function on each component are given in the appendix (p 2) and were based on the report by Fried and colleagues.^1^ Frailty was defined by the presence of at least three frailty components and pre-frailty defined by one or two frailty components.

### Socioeconomic status

Occupational class of each participant was based on the current or most recent Civil Service employment grade, at age 45–55 years. Employment grade characterised classes of individuals with similar income, pension rights, job security, and work skills, and was divided into high, intermediate, and low groups.

### Behavioural and biomedical risk factors

Risk factor levels were based on measurements made at age 45–55 years. Behaviours (smoking, alcohol intake, physical activity, and fruit and vegetable intake) and biomedical risk factors (body-mass index, serum cholesterol, serum high-density lipoprotein (HDL) cholesterol, fasting plasma glucose, serum interleukin-6 [IL-6] and C-reactive protein) were derived from self-reports and clinical data.^20^, ^21^ Participants were assigned to three physical activity groups: inactive; less than 1 h per week of moderate and vigorous activity, 2·5 h or more per week of moderate activity, or 1 h or more of vigorous activity; and moderately active. For lung function, we used the largest forced expiratory volume (FEV) in 1 s (measured in L) value of three attempts.^22^ Lung volumes are related to body size, therefore we corrected FEV for height by dividing by the square of the participant's standing height and multiplying by the square of the sample mean height, 1·77 m in men and 1·63 m in women, so that variation in lung function was due to factors other than body size.^23^

### Disease status and disability

Disease status was identified at age 45–55 years. Depressive symptoms were defined according to general health questionnaire caseness (GHQ-30, score ≥5), hypertension of at least 140 mm Hg systolic or at least 90 mm Hg diastolic blood pressure or on antihypertensive treatment, diabetes based on a standard 75 g oral glucose tolerance test or self-reported doctor diagnosis, and cardiovascular disease as non-fatal myocardial infarction or definite angina according to self-report, clinical data, and health records. Disability was defined as one or more impairments of six self-care tasks on the Activities of Daily Living (ADL) questionnaire: dressing, walking across the room, bathing or showering, eating, getting in or out of bed, and using the toilet.^16^

### Statistical analysis

The study sample was defined as those who participated at the fifth (2007–09), sixth (2012–13), or seventh (2015–16) clinics and in whom frailty was assessed at least once. For each level of each risk factor, we calculated the number of individuals, total person-observations, and cases of frailty, together with the percentage of all observations that were considered cases of frailty. For each employment grade, we also calculated the percentage of observations that were cases of frailty from observations that fulfilled at least one of the five frailty conditions. Additionally, of the observations that were not cases of frailty, we calculated the percentage of observations that were cases of pre-frailty.

We used multiple imputation for missing risk factor data, replacing missing values with values drawn randomly from the predictive distribution of each risk factor conditional on the observed data as specified by an imputation model. This process created multiple imputed datasets that each had no missing values and accounted for the uncertainty due to the missing data. The imputation model contained all assessments of the participants' frailty status together with all the risk factors at age 50 years as described previously.^16^ Additionally, the following auxiliary variables, which were associated with at least one of the risk factors with missing data, were included in the imputation model: data wave when participant was aged 50 years; values of all the risk factors at ages below and above age 50 years from adjacent data collections; waist circumference; prevalent angina, myocardial infarction, stroke, and systolic and diastolic blood pressure; family history of high blood pressure; and satisfaction with standard of living. We generated ten imputed datasets that were analysed separately and combined the results using Rubin's rules.^24^

We fitted logistic mixed models for frailty to estimate the associations between each risk factor at age 50 years and subsequent assessments of the frailty outcome. These models made use of data from all available clinic visits when frailty was measured while also accounting for differences in the timing of these occasions and the correlated nature of the data from repeated measures of frailty from the same individual over time. For those attending the fifth clinic (2007–09), when frailty was first assessed, age was defined at the date of attendance. The timing of frailty measurements at the sixth and seventh clinics was accounted for by use of the interval since the fifth clinic as the time variable. For those who did not attend the fifth clinic, the midpoint date of that clinic was used to calculate age and the time variable. A random intercept was fitted to allow for individual differences in frailty at the fifth clinic.

The base model was initially adjusted for age and age squared at the fifth clinic, time of frailty measurement since the fifth clinic, sex, and ethnic origin to estimate odds ratios of frailty for each level of the risk factor relative to a reference level that was considered healthy. For the biomedical factors, we estimated the odds ratio of frailty per one standard deviation increase in each factor. To assess the potential contribution of each risk factor to the trend of increased frailty with lower employment grade, we added each factor separately into a base model, adjusted with the addition of a separate marital status term for each sex and employment grade and calculated the percentage change in the employment grade gradient compared with the base model. Furthermore, we adjusted for groups of risk factors a priori, and then for all risk factors together, to assess the amount of attenuation of the employment grade gradient. Predictors of impaired functioning in a previous study^16^ (lung function, hypertension, physical inactivity, and inflammatory markers) were also grouped for adjustment.

We completed sensitivity analyses with logistic regression and multiple imputation for missing risk factor values, using individuals rather than person-observations as the unit of analysis, by examining the associations of employment grade and the risk factors with ever having frailty one or more times, as assessed from the three clinics in 2007–09, 2012–13, and 2015–16. With these models we used bootstrap methods to compute 95% CI for the percentage attenuation in the employment grade effects.

We used the methods of Fine and Gray^25^ to adjust for the competing risk of attrition from our study before the seventh clinic by use of individuals as the unit of analysis in a time-to-event framework, incorporating a modification^26^ that reallocated participants with attrition to frailty cases according to their probability of becoming frail during the follow-up period. All analyses were done in SAS version 9.4.

### Data sharing

Whitehall II data are available to bona fide researchers for research purposes. Please refer to the Whitehall II data sharing policy. Definition of variables and statistical codes are provided in the appendix (pp 3–5).

### Role of the funding source

The funders of the study had no role in study design, data collection, data analysis, data interpretation, or writing of the report. The corresponding author had full access to all the data in the study and had final responsibility for the decision to submit the manuscript for publication.

## Results

**Figure 1:**
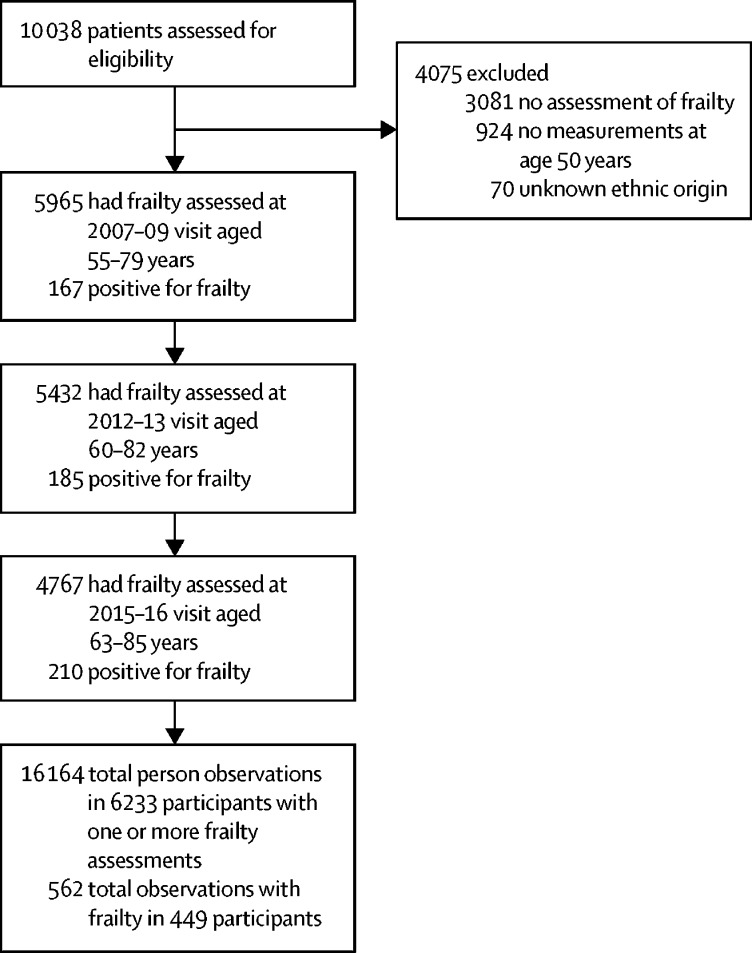
Study profile Full information about the number and proportion of participants with frailty measurements at each clinic is provided in the appendix (p 6).

**Figure 2:**
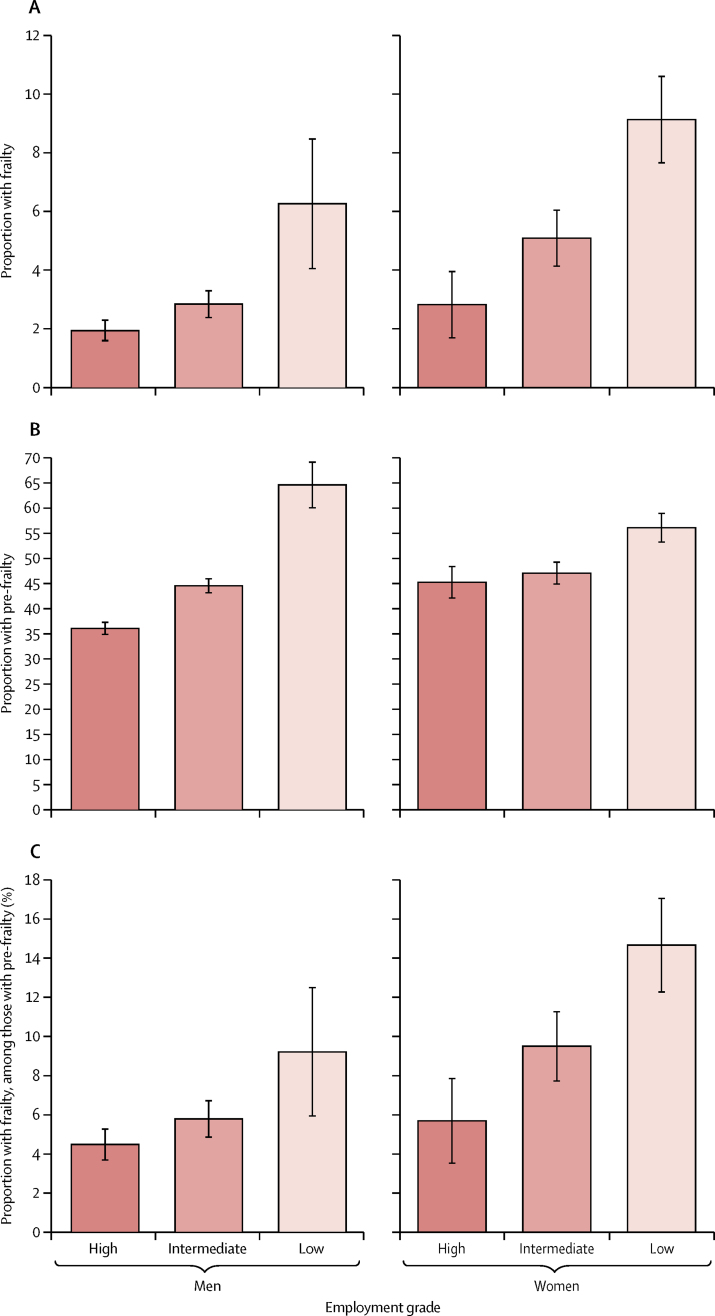
Age-standardised frailty and pre-frailty observations by employment grade and sex Error bars show 95% CI. Figure shows age-standardised frailty (A) and pre-frailty (B) by employment grade and sex as a proportion of person-observations in the total population, and age-standardised frailty as a proportion of person-observations in those meeting at least one of the five frailty components (ie, excluding non-frail person-observations; C). Tests for trend by employment grade were all p<0·0001 except for men in C, which was p=0·0026.

In 1985, 10 308 civil servants were entered into the Whitehall II study (figure 1); the maximum follow-up was from 1985 to 2016, with the median follow-up from age 50 years being 18·0 years (IQR 14·5–22·8). Between Sept 9, 2007, and Dec 8, 2016, frailty was assessed and found to be present in 562 (4%) of 16 164 person-observations at mean age 69 years (SD 5·9) and varied by socioeconomic status: 145 (2%) person-observations had high socioeconomic status, 241 (4%) had intermediate status, and 176 (7%) had low socioeconomic status. Frailty was also associated with older age, female sex, and non-white ethnic origin at age 50 years (table 1). Prevalence of the components of frailty, other than weight loss, increased with lower employment grade in the whole sample (appendix p 7). Among the 562 person-observations with frailty, the only component showing a social gradient was walking speed in women (appendix p 8). In both sexes, there were gradients according to employment grade in the proportion of frailty and pre-frailty overall, and in the proportion of frailty among person-observations defined as pre-frail or frail (figure 2). Disability, defined as one or more impairments as defined on the ADL questionnaire, was present more often in observations of frail individuals (278 [47%] of 594 person-observations) than in observations of non-frail or pre-frail individuals (1382 [9%] of 15 433 person-observations).

**Table 1:** Presence of frailty at 2007–09, 2012–13, or 2015–16 visits, by sociodemographic characteristics in 6233 participants

		**Individuals**	**Person-observations**	Positive observations for frailty adjusted for sex and age (%)*	**Odds ratio**†**(95% CI)**
Total sample		6233	16 164	562 (4%)	..
Age group at fifth clinic (2007–09), years					
	≤64	3235	8695	150 (2%)	Ref (1·0)
	65–69	1348	3526	128 (4%)	1·98 (1·51–2·59)
	70–74	1269	3102	193 (6%)	3·70 (2·89–4·74)
	≥75	381	841	91 (11%)	7·55 (5·44–10·5)
Sex					
	Male	4456	11 691	289 (3%)	Ref (1·0)
	Female	1777	4473	273 (6%)	2·42 (1·98–2·96)
Ethnic origin					
	White	5734	14 979	455 (3%)	Ref (1·0)
	South Asian	289	692	67 (8%)	2·95 (2·10–4·15)
	Black	166	379	27 (5%)	1·91 (1·17–3·11)
	Other	44	114	13 (9%)	3·58 (1·67–7·68)
Marital status					
	Married or cohabiting	4662	12 198	341 (3%)	Ref (1·0)
	Single, divorced, or widowed	1253	3171	195 (6%)	2·13 (1·71–2·65)
	Missing	318	796	26	..
Socioeconomic status (last known grade at age 50 years)					
	High	2677	7204	145 (2%)	Ref (1·0)
	Intermediate	2764	7119	241 (4%)	1·48 (1·16–1·88)
	Low	792	1841	176 (7%)	2·60 (1·89–3·58)

Data are n (%).

* Age-adjusted (at the fifth clinic) and sex-adjusted prevalence of frailty for all characteristics. Values for sex are age-adjusted.

† Odds ratios for each risk factor after multiple imputation for missing values, adjusted for age and age squared at the fifth clinic, time of frailty measure since fifth clinic, sex, and ethnic origin.

After adjustment for age and sex, predictors of frailty at age 50 years included abstinence or high consumption of alcohol, current smoking status, low daily fruit and vegetable consumption, moderate or no physical activity, low lung function (measured by FEV), overweight and obesity, depressive symptoms, hypertension, and cardiovascular disease (table 2); being an ex-smoker, underweight, or having a diagnosis of diabetes did not significantly affect frailty risk. Blood biomarker risk factors for frailty (measured at age 50 years) were low HDL cholesterol, low ratio of total to HDL cholesterol, and high concentrations of IL-6 and C-reactive protein (table 3).

**Table 2:** Presence of frailty at 2007–09, 2012–13 or 2015–16 by risk factors measured at age 50 years

	**Individuals (n=6233)**	**Person-observations (n=16 164)**	Positive observations for frailty adjusted for sex and age (%)*	**Odds ratio**†**(95% CI)**
**Smoking status**				
Never smoker	2972	7870	292 (4%)	Ref (1·0)
Ex-smoker	2211	5782	152 (3%)	0·85 (0·67–1·07)
Current smoker	679	1615	92 (5%)	1·69 (1·27–2·25)
Missing	371	897	26	..
**Alcohol consumption**				
None	893	2218	147 (5%)	1·85 (1·43–2·38)
Moderate (women: ≤ 14 units per week; men: ≤21 units per week)	3898	10 216	297 (3%)	Ref (1·0)
High	1132	2981	91 (4%)	1·54 (1·17–2·04)
Missing	310	749	27	..
**Fruit and vegetable consumption**				
≥ Daily	3921	10 348	335 (3%)	Ref (1·0)
< Daily	2011	5097	206 (4%)	1·29 (1·05–1·58)
Missing	301	719	21	..
**Physical activity**				
Active	3274	8584	202 (3%)	Ref (1·0)
Moderately active	1307	3421	130 (4%)	1·52 (1·17–1·97)
Inactive	1258	3216	195 (6%)	2·63 (2·06–3·37)
Missing	394	943	35	..
**FEV (adjusted for height)**				
Tertile 1 (lowest; <2·91 L)	291	783	19 (2%)‡	1·90 (1·36–2·65)
Tertile 2 (2·91–3·58 L)	297	829	10 (1%)	1·54 (1·25–1·90)
Tertile 3 (highest; >3·58 L)	299	843	4 (1%)	Ref (1·0)
Missing	5346	13 709	529	..
Per 1SD increase	..	..	..	0·63 (0·55–0·72)
**Adiposity**				
Underweight (<18·5 kg/m^2^)	38	102	5 (5%)	1·95 (0·67–5·66)
Normal weight (18·5–24·9 kg/m^2^)	2796	7363	217 (3%)	Ref (1·0)
Overweight (25·0–25·9 kg/m^2^)	2173	5628	190 (3%)	1·34 (1·07–1·69)
Obese (≥ 30·0 kg/m^2^)	578	1444	117 (8%)	3·52 (2·62–4·72)
Missing	648	1627	33	..
**Depressive symptoms**				
No	4377	11 349	336 (3%)	Ref (1·0)
Yes	1542	4062	198 (5%)	1·65 (1·33–2·03)
Missing	314	753	28	..
**Hypertension**				
No	4645	12 226	408 (3%)	Ref (1·0)
Yes	1169	2944	137 (5%)	1·39 (1·10–1·76)
Missing	419	994	17	..
**Diabetes**				
No	6063	15 746	544 (3%)	Ref (1·0)
Yes	170	418	18 (5%)	1·62 (0·92–2·88)
**Cardiovascular disease**				
No	6091	15 819	543 (3%)	Ref (1·0)
Yes	142	345	19 (8%)	2·11 (1·18–3·79)

Data are n (%). FEV=forced expiratory volume.

* Age-adjusted (at the fifth clinic) and sex-adjusted prevalence of frailty, except for FEV for which unadjusted prevalence is shown.

† Odds ratios for each risk factor after multiple imputation for missing values, adjusted for age and age-squared at the fifth clinic, time of frailty measure since fifth clinic, sex, and ethnic origin in 6233 participants.

‡ For FEV tertiles, unadjusted prevalence is shown because the age range of this sample at the fifth clinic was restricted (all ages ≤64 years).

**Table 3:** Presence of frailty at 2007–09, 2012–13 or 2015–16 by biomedical factors measured at age 50 years

	**Individuals (n=6233)**	**Person-observations (n=16 164)**	Positive observations for frailty adjusted for sex and age (%)*	**Odds ratio (95% CI)**†
**Total cholesterol, mmol/L**				
Tertile 1 (<5·60)	1875	4992	152 (3%)	Ref (1·0)
Tertile 2 (5·60–6·50)	2007	5247	195 (4%)	1·07 (0·84–1·38)
Tertile 3 (>6·50)	1914	4887	191 (3%)	1·04 (0·81–1·34)
Missing	*437*	1038	24	..
Per SD increase	..	..	..	1·03 (0·93–1·14)
**HDL cholesterol, mmol/L**				
Tertile 1 (<1·25)	1453	3855	103 (4%)	1·57 (1·16–2·12)
Tertile 2 (1·25–1·59)	1383	3695	113 (4%)	1·28 (0·96–1·71)
Tertile 3 (>1·59)	1492	4002	117 (3%)	Ref (1·0)
Missing	1905	4612	229	..
Per SD increase	..	..	..	0·81 (0·71–0·93)
**Total cholesterol: HDL cholesterol ratio**				
Tertile 1 (<1·31)	1442	3883	110 (3%)	Ref (1·0)
Tertile 2 (1·31–1·57)	1442	3870	95 (4%)	1·08 (0·78–1·50)
Tertile 3 (>1·57)	1442	3793	128 (5%)	1·64 (1·22–2·19)
Missing	1907	4618	229	..
Per SD increase	..	..	..	1·20 (1·07–1·36)
**Fasting glucose, mmol/L**‡				
Tertile 1 (<4·90)	1159	3162	68 (2%)	Ref (1·0)
Tertile 2 (4·90–5·29)	1271	3457	71 (3%)	1·07 (0·72–1·58)
Tertile 3 (>5·29)	1245	3373	68 (3%)	1·12 (0·80–1·57)
Missing	2558	6172	355	..
Per SD increase	..	..	..	1·04 (0·95–1·14)
**Interleukin-6 concentration, pg/mL**				
Tertile 1 (<1·06)	1245	3570	32 (1%)	Ref (1·0)
Tertile 2 (1·06–1·63)	1215	3372	82 (3%)	1·73 (1·23–2·44)
Tertile 3 (>1·63)	1237	3088	103 (4%)	2·23 (1·59–3·13)
Missing	2536	6134	345	..
Per SD increase	..	..	..	1·41 (1·26–1·57)
**C-reactive protein concentration, mg/L**				
Tertile 1 (<0·56)	1235	3581	50 (2%)	Ref (1·0)
Tertile 2 (0·56–1·37)	1229	3382	56 (2%)	1·14 (0·83–1·56)
Tertile 3 (>1·37)	1245	3096	110 (4%)	1·94 (1·47–2·56)
Missing	2524	6105	346	..
Per SD increase	..	..	..	1·36 (1·21–1·53)

Data are n (%). HDL=high-density lipoprotein.

* Age-adjusted (at the fifth clinic) and sex-adjusted prevalence of frailty.

† Odds ratios of frailty associated with each biomedical factor after multiple imputation for missing values, adjusted for age and age squared at fifth clinic, time of frailty measure since fifth clinic, sex, and ethnic origin, in 6233 participants.

‡ Fasting glucose was measured in patients without diabetes only.

We examined the nature of the association of last known employment grade at age 50 years with frailty. The effect of adjustment of a base model for each potential contributing factor was analysed in turn (table 4). Comparison of the prevalence of risk factors by employment grade at the start of the study in the analytic sample, and those excluded from the sample, revealed evidence of health-related selection but similar risk factor trends by grade (appendix p 9). Attenuation of the grade–frailty association was more than 4% after model adjustment for smoking status, alcohol consumption, lung function as measured by FEV, body-mass index category, HDL cholesterol concentration, ratio of total cholesterol to HDL cholesterol, and IL-6 and C-reactive protein concentrations. Risk factor attenuations were similar in men and women except for alcohol consumption, physical activity, and lung function (appendix p 10), with alcohol accounting for greater attenuation in women and physical activity and FEV accounting for greater attenuation in men.

**Table 4:** Effect of adjustment of the base model for each potential contributing factor on the association of frailty at 2007–09, 2012–13, or 2015–16, with last known employment grade at age 50 years

	Odds ratio (95% CI)*	**Percent change**†
Base model‡	1·49 (1·27–1·75)	Ref (1·00)
Base model + smoking status	1·46 (1·24–1·72)	−4·6%
Base model + alcohol consumption	1·45 (1·23–1·70)	−7·0%
Base model + frequency of fruit or vegetable consumption	1·47 (1·25–1·73)	−2·8%
Base model + physical activity	1·41 (1·20–1·66)	−13·4%
Base model + FEV	1·43 (1·22–1·68)	−10·0%
Base model + body-mass index category	1·42 (1·21–1·68)	−11·0%
Base model + depressive symptoms	1·51 (1·28–1·77)	+3·5%
Base model + hypertension	1·49 (1·27–1·75)	+0·6%
Base model + diabetes	1·48 (1·26–1·74)	−0·6%
Base model + cardiovascular disease	1·48 (1·26–1·74)	−1·1%
Base model + total cholesterol	1·49 (1·27–1·75)	+0·1%
Base model + HDL cholesterol	1·45 (1·23–1·71)	−6·3%
Base model + total cholesterol: HDL ratio	1·46 (1·24–1·72)	−4·7%
Base model + fasting glucose§	1·50 (1·27–1·77)	+1·6%
Base model + interleukin-6 concentration	1·41 (1·20–1·66)	−13·0%
Base model + C-reactive protein concentration	1·43 (1·21–1·68)	−10·6%

FEV=forced expiratory volume in 1 s.

* Odds ratios of frailty from trend with last known grade on one degree of freedom (ie, the odds ratio of frailty for one unit lower grade level, across low, intermediate, and high employment grades) in 6233 participants.

† Percentage change in coefficient (log odds ratio) for trend across employment grade, compared with the base model. A negative change indicates attenuation of the social gradient compared with the base model.

‡ Base model is adjusted for age and age squared at fifth clinic, time of frailty measure since fifth clinic, sex, ethnic origin, marital status, and marital status by sex interaction.

§ Fasting glucose was measured in patients without diabetes only.

Table 5 shows the effect of adjustment for six groups of risk factors. Adding body-mass index to the behavioural group of risk factors increased the attenuation of the socioeconomic gradient in frailty from 23% to 30%. Adjustment for prevalent disease at age 50 years (ie, cardiovascular disease, diabetes, and depressive symptoms) had little effect on the association. Adjustment for inflammatory markers as well as hypertension, physical activity, and lung function increased attenuation of the gradient in frailty from 22% to 33%. Adjustment for all covariates produced an attenuation of 39%. Sensitivity analysis showed physical activity but not body-mass index contributed substantially to the attenuation when removed from the adjustment. Attenuation on adjustment for groups of risk factors tended to be larger in men than women (appendix p 11). There was no interaction of the frailty effects of employment grade and the other risk factors with age.

**Table 5:** Effect of adjustment for potential contributing factors on the association of frailty at 2007–09, 2012–13, or 2015–16 visits with trend in last known employment grade at age 50 years

		**Model adjustments**	Odds ratio*(95% CI)	**Percent change**†
Base model		Age at fifth clinic, age squared, sex, ethnic origin, marital status, marital status by sex interaction, and interval between the fifth clinic visit and subsequent visits when frailty was measured	1·49 (1·27–1·75)	Ref (1·00)
Health behaviours		Base model plus smoking status, alcohol use, physical activity, and fruit or vegetable consumption	1·36 (1·15–1·61)	−22·9%
Health behaviours plus body-mass index		Base model plus smoking status, alcohol use, physical activity, fruit or vegetable consumption, or body-mass index	1·32 (1·11–1·56)	−30·4%
Disease status		Base model plus prevalent cardiovascular disease, diabetes, and depressive symptoms	1·50 (1·27–1·76)	+1·7%
Predictors of impaired functioning				
	Cognitive and physical functioning	Base model plus hypertension, physical activity, and FEV	1·36 (1·16–1·61)	−21·6%
	Cognitive, physical functioning, and inflammatory markers	Base model plus hypertension, physical activity, FEV, and inflammatory markers (C-reactive protein and interleukin-6)	1·30 (1·10–1·54)	−33·4%
All covariates		Base model plus smoking status, alcohol use, physical activity, fruit or vegetable consumption, body-mass index, hypertension, physical activity, FEV, inflammatory markers, HDL, prevalent cardiovascular disease, diabetes, and depressive symptoms	1·28 (1·07–1·51)	−38·7 %
Sensitivity analysis				
	All covariates, excluding physical activity	Base model plus smoking, alcohol use, fruit or vegetable consumption, body-mass index, hypertension, FEV, inflammatory markers, HDL, prevalent cardiovascular disease, diabetes, and depressive symptoms	1·31 (1·10–1·56)	−31·7%
	All covariates, excluding body-mass index	Base model plus smoking, alcohol use, physical activity, fruit or vegetable consumption, hypertension, FEV, inflammatory markers, HDL, prevalent cardiovascular disease, diabetes, and depressive symptoms	1·28 (1·08–1·52)	−37·3%
	All covariates, excluding physical activity and body-mass index	Base model plus smoking, alcohol use, fruit or vegetable consumption, hypertension, FEV, inflammatory markers, HDL, prevalent cardiovascular disease, diabetes, and depressive symptoms	1·32 (1·11–1·57)	−29·7%

FEV=forced expiratory volume in 1 s. HDL=high-density lipoprotein.

* Odds ratios of frailty from trend with last known grade on one degree of freedom (ie, the odds ratio of frailty for one unit lower grade level, across low, intermediate, and high employment grades) in 6233 participants.

† Percentage change in coefficient (log odds ratio) for trend across employment grade, compared with the base model.

Further sensitivity analysis with logistic regression using individuals, rather than person-observations, as the unit of analysis produced similar findings for the frailty gradient and risk factor attenuations whether adjusted for singly or in groups (appendix pp 12–13). Bootstrap 95% CIs were estimated for the percent changes in the employment grade effects in this mode of analysis. Generally, an attenuation of more than 4% was associated with a confidence interval that excluded the null, and thus a significant attenuation. The competing risks analysis suggested the trend in frailty by employment grade was slightly underestimated as a consequence of sample attrition. There were 282 deaths among non-frail participants before the seventh clinic (2015–16) when frailty was last assessed. Additionally, 1035 non-frail participants were not assessed after the fifth (2007–09) or sixth (2012–13) clinic. The proportion of loss to follow-up by employment grade differed considerably for drop-out but not for death (appendix p 14). We reassigned 111 (8%) drop-outs from the non-frail group to the frail category on the basis of a propensity score of more than 67% of the scores of observed frailty cases. The trend in frailty on employment grade was increased by 5%.

## Discussion

Socioeconomic status shapes midlife exposure to many risk factors for poor health.^27^, ^28^ Accordingly, in this prospective cohort study, we found a socioeconomic gradient in frailty at ages 55–85 years, defined on the basis of occupation at age 50 years. Health behaviours and biomedical risk factors measured at age 50 years accounted for more than a third of socioeconomic inequality in frailty. The five most important contributing factors that individually accounted for 10% or more of the socioeconomic gradient in frailty were physical activity level, lung function by spirometry, body-mass index category, and serum IL-6 and C-reactive protein concentrations. These observations provide evidence that midlife might be an appropriate age for public health and clinical interventions intended to reduce socioeconomic inequality in the functional health of older people as well as to reduce the number of frail older people. The analyses provide an estimate, making assumptions about reversibility of the risks, of the upper bound for attenuation of the social gradient in frailty that might be possible as a result of intervening on the risk factors identified.

We previously determined the associations of risk factors at age 50 years for impaired physical and cognitive functioning at older ages in the same setting.^16^ Physical inactivity, hypertension, and poor lung function stood out as potential midlife intervention targets to reduce likelihood of both types of functional impairment in old age. In this study, we used frailty, an outcome that is functionally related to but distinct from the Mini-Mental State Examination and ADL instruments used in our previous analysis. Our novel analysis with 18 years' median follow-up confirms the importance of physical inactivity, hypertension, and poor lung function in midlife as risk factors for poor functional health at older ages.

Of the different frailty operationalisations, we used the well-known standard frailty phenotype,^29^ identified at one or more of three follow-up visits. The phenotype is an index of physical functioning, low vitality (ie, exhaustion), and recent weight loss; cognitive function is not included in the definition. We observed the expected association between frailty and disability. Low energy expenditure from physical activity is one of the five frailty criteria, and (as expected) physical activity level, particularly physical inactivity, at age 50 years was a risk factor for frailty.^16^, ^30^ Physical activity level at age 50 years is also an important contributor to the socioeconomic difference in frailty, reflecting the association of inactivity with lower employment grade and conversely of higher physical activity with higher grade in midlife in the present study. The social gradient in physical activity level continues at older ages but the inverse trend in frailty occurrence across socioeconomic strata is not generated by the physical activity component. Some 90% of frailty cases met the physical inactivity criterion, the distribution of which among cases did not differ by employment grade.

Likewise, weight loss is one of the frailty criteria, and body-mass index at age 50 years is a risk factor. Potentially, high body-mass index in midlife, and associated later weight loss, could be responsible for distortion of frailty occurrence due to regression to the mean. However, few person-observations met the weight-loss criterion (loss >10% over 5 years) for frailty at follow-up and there was an even distribution of weight loss, thus defined, among frailty cases across employment grades in men and women.

Frailty can accompany or be a precursor to partial or total dependence on caregiver support.^10^, ^11^, ^13^, ^15^, ^31^ Reversing damage to the physiological systems underlying the frailty phenotype might not be feasible once it has developed. For example, poor lung function in chronic obstructive pulmonary disease is at best partly reversible.^32^ In the present study, ex-smokers at age 50 years did not have increased odds of frailty compared with never-smokers, but current smokers did have higher odds of frailty. Thus, lung function is an important target of intervention in early adulthood and midlife, whereas increased physical activity is likely to yield benefits for health-related functioning, including mobility, among those able to adopt it, even at older ages. Both overweight and obesity at age 50 years increased the odds of frailty at older ages.^33^ It could be that intentional weight loss, as a result of calorie restriction or increased physical activity, is protective for frailty. By contrast, unintentional loss of weight is one of the five components defining frailty, and is fairly common in older adults.^34^

The key variables accounting for the socioeconomic gradient in frailty differed partly from those found to be risk factors for frailty overall. Physical inactivity, low FEV, and raised body-mass index were important mediators of the gradient as well as having main effects. Adjusting for IL-6 and C-reactive protein as well as hypertension, physical inactivity, and low FEV, led to further marked attenuation of the socioeconomic gradient in frailty, in line with the role of serum C-reactive protein concentration in predicting ADL disability.^16^ Although hypertension was a risk factor for frailty, controlling for it scarcely changed the coefficient for employment grade. Likewise, depression and cardio-vascular disease status at age 50 years were frailty risk factors but neither accounted for its socioeconomic gradient. Smoking at age 50 years both was a risk factor for frailty and accounted for 5% of the gradient in frailty. As noted above, in terms of frailty risk reduction in midlife, risk was similar among ex-smokers and never-smokers at age 50 years.

We found that about a third of socioeconomic inequality in frailty at older ages was accounted for by health behaviours and cardiovascular risk factors measured at age 50 years. The analysis was based on a relatively privileged population sample, largely composed of office-based civil servants.^18^ Although their health status is better than that of participants in a general non-occupational cohort in terms of mortality, morbidity, and risk factor distributions, causal associations between risk factors and incidental disease are generalisable.^35^ The analytical sample had favourable proportions of risk factors compared with those who dropped out or died before providing a frailty assessment. Associations of risk factors with employment grade were broadly similar. Considering the absence of manual workers in the Whitehall II cohort, perhaps the proportion of the socioeconomic gradient in frailty estimated by adjusting for the risk factors we studied (roughly a third) would be larger in the general population. This statistical approach is equivalent to standardising the risk factor distributions across the sample, and does not take account of the effectiveness of intervention in practice.

How is the unexplained proportion of the frailty gradient to be understood? First, we focused on midlife behaviours, chronic disease, and biomedical risk factors, although risk and protective factors in early life might also have a role in socioeconomic differences in frailty. Neither childhood socioeconomic circumstances nor health-related social selection were examined; however, both pathways have been shown to be modest in size compared with adult social causation.^27^, ^36^ Depressive symptoms in this cohort did not contribute to the social gradient in frailty, and other midlife psychosocial risk factors such as job stress were not analysed.^8^, ^37^, ^38^, ^39^ Second, exposure was assessed at a single point in midlife and biological risk accumulation over the life course was therefore measured suboptimally.^40^, ^41^ Third, changes in disease and risk factor status before exposure assessment or after exposure assessment and before onset of frailty were not included in the analysis. Further research on the potential of modifiable early-life and old-age factors to reduce the socioeconomic gradient in frailty is needed.

The study has several defining characteristics. We did a prospective analysis of the associations of employment grade, and behavioural, biomedical, and disease exposures around age 50 years with occurrence of frailty approximately 18 years later. The inverse association of lower employment grade with risk of frailty was seen across the whole age range (55–85 years). Some risk factor effects on frailty were marginally weaker in older participants; however, these differences were small and there were no significant interactions with age. Missing risk factor data, but not outcome data, were filled in using multiple imputation. This procedure assumes the missing data are missing at random. The overall prevalence of frailty was 3%, which is close to the level in the corresponding age group in the Cardiovascular Health Study^1^ used to develop the definition of the frailty phenotype. Competing risks analysis, taking account of differing rates of death and loss to follow-up by employment grade in non-frail individuals, indicated that the observed socioeconomic gradient in frailty was slightly attenuated by sample attrition.

In conclusion, this study sought to quantify the potential contribution of modifiable risk factors at age 50 years to later-life inequalities in frailty. Risk factors for socioeconomic inequality in frailty differed from those for frailty in the whole sample. For example, hypertension was a predictor of frailty overall but did not contribute to frailty inequality. Behaviour-related factors accounted for 30% of the socioeconomic gradient, particularly smoking, alcohol consumption, physical activity, fruit and vegetable consumption, and body-mass index. Addition of lung function and inflammatory markers to this group of predictors attenuated the socioeconomic gradient by a further third, to 40%. Our findings suggest that frailty, and socioeconomic inequality in frailty, could at least partly be avoidable. Further research is needed to address other factors, including perceived control over health,^39^ that might explain social differences in this important measure of wellbeing in older people.
