# Supplementary material for: Midlife contributors to socioeconomic differences in frailty during later life: a prospective cohort study
Source: Lancet Public Health. 2018 Jun 14;3(7):e313–22. doi: 10.1016/S2468-2667(18)30079-3 (PMC6120440; doi:10.1016/S2468-2667(18)30079-3)
Supplement: Supplementary appendix [file mmc1.pdf]

THE LANCET
Public Health

Supplementary appendix

This appendix formed part of the original submission and has been peer reviewed. We post it as supplied by the authors.

Supplement to: Brunner EJ, Shipley MJ, Ahmadi-Abhari S, et al. Midlife contributors to socioeconomic differences in frailty during later life: a prospective cohort study. *Lancet Public Health* 2018; published online June 13. http://dx.doi.org/10.1016/S2468-2667(18)30079-3.

APPENDICES

Midlife contributors to socioeconomic differences in later life frailty: prospective study

Brunner EJ, Shipley MJ, Ahmadi-Abhari S, Valencia Hernandez C, Abell J, Singh-Manoux A, Kawachi I, Kivimaki M

Item	Appendix/Figure/Table	Page
1	Components used in the Fried frailty scale	2
2	Specification of variables used and fitted models	3
3	Figure A1: Number and proportion of 6233 participants with 1, 2 and 3 frailty measurements by clinic	6
4	Table A1: Age standardised prevalence (%, and 95% confidence interval) of the components of frailty by employment grade among 16164 person-observations, by sex	7
5	Table A2: Age standardised prevalence (%) by employment grade of the components of frailty among 562 person-observations with frailty, by sex	8
6	Table A3: Comparison of the prevalence of risk factors in participants in the analytic sample and those excluded, and their associations with employment grade measured at the start of the Whitehall II study (1986-1988).	9
7	Table A4: Effect of adjustment for each potential contributing factor on the association of frailty at 2007-2009, 2012-2013 or 2015-2016 with trend in last known employment grade at age 50, in men and women	10
8	Table A5: Effect of adjustment for potential contributing factors on the association of frailty at 2007-2009, 2012-2013 or 2012-2013 with trend in last known employment grade at age 50, in men and women	11
9	Table A6: Effect of adjustment for each potential contributing factor on the association of ever having frailty at 2007-2009, 2012-2013 or 2015-2016 with last known employment grade at age 50 among 6233 participants	12
10	Table A7: Effect of adjustment for potential contributing factors on the association of ever having frailty at 2007-2009, 2012-2013 or 2015-2016 with last known employment grade at age 50 among 6233 participants	13
11	Table A8: Frailty and mortality outcomes and attrition by employment grade among 6233 participants	14

Components used in the Fried frailty scale

1. *Walking speed*: based on the time taken to walk a distance of 8 feet (2.4 m) at usual pace. Established cut-offs for the slow walking speed criterion for frailty have been based on the time taken to walk a distance of 15 feet (4.6 meters). Accordingly, participants were categorized as having slow walking speed when time to walk 8 feet was ≥ 3.73 seconds (for men with height ≤ 173 cm or women with height ≤ 159 cm) or ≥ 3.20 seconds (for men with height > 173 cm or women with height > 159 cm).

2. *Grip strength*: measured in kilograms using the Smedley hand grip dynamometer. Cut-offs were stratified by gender and body mass index (BMI). For men, low grip strength was denoted as: ≤ 29 kg (BMI ≤24 kg/m^2), ≤ 30 (BMI 24.1-28), and ≤ 32 (BMI > 28). For women, low grip strength was: ≤ 17 (BMI ≤23), ≤ 17.3 (BMI 23.1-26), ≤ 18 (BMI 26.1-29), and ≤ 21 (BMI > 29).

3. *Weight loss*: weight loss in the context of frailty has been defined as unintentional weight loss of ≥5% body weight lost over the previous year. This same measure was not available in the Whitehall II study. For each of the phases 9, 11 and 12, we calculated percentage weight loss since the previous phase (7, 9 and 11 respectively), and used weight loss >10% as the weight loss criterion for frailty, in accordance with that in the Women's Health Aging Study-I.

4. *Exhaustion*: defined using two items drawn from the Center for Epidemiology Studies-Depression (CES-D) scale: "I felt that everything I did was an effort in the last week" and "I could not get going in the last week". If participants answered "occasionally or moderate amount of the time (3-4 days)" or "most or all of the time (5-7 days)" to either of these items, they were categorized as exhausted.

5. *Physical activity*: based on a modified version of the Minnesota leisure-time physical activity questionnaire which includes 20 items on the frequency and duration of participation in different physical activities (e.g., running, cycling, other sports, housework, and gardening activities). Total hours per week were calculated for each activity and a metabolic equivalent (MET) value was assigned to each based on a compendium of values. Energy expenditure (kcal/week) was computed for each participant. Low levels of physical activity were denoted by an expenditure of <383 kcal/week in men and <270 kcal/week in women.

A total frailty score was calculated by allocating a value of 1 to each of the above criteria if present, resulting in a range of 0 to 5. Participants were classified as "frail" if they had at least three out of the five frailty components; as "pre-frail" if they had 1 or 2 components; and as "non-frail" if they had none of these components.

Specification of variables used and fitted models

Variable	Description or coding
id	Subject identifier
frailty2	Frailty (non-frail or pre-frail = 0, frail = 1)
age	Age(years) at fifth clinic (first frailty measurement) in 2007-2009
agegroup	Factor for age group at fifth clinic (first frailty measurement) in 2007-2009
time	Time(years) since fifth clinic
ethnicity	Factor with 4 groups (white, south asian, black, other)
riskfactor	Risk factor of interest (continuous variable or a factor)
mstatus	Marital status at age 50 (not married =0, married/cohabiting = 1)
ses	Employment grade at age 50 (continuous variable: high = 1, intermediate = 2, low = 3)
sesfactor	Factor for employment grade at age 50 (high = 1, intermediate = 2, low = 3)
imputation	Variable to identify the 10 imputed datasets

Models for Table 1

```
proc glimmix data=<10 datasets with missing values imputed> noclprint order=formatted;
 model frailty2 (event='1') = agegroup sex ethnicity time
                                  / solution dist=binary link=logit ;
 random intercept / subject = id ;
 by   _imputation_  ;
 nloptions tech=newrap ;
 ods output ParameterEstimates=outests ;
run;
proc mianalyze parms=outests;
 modeleffects agegroup ;
run;

proc glimmix data=<10 datasets with missing values imputed> noclprint order=formatted;
 model frailty2 (event='1') = age age^2 sex ethnicity time
                                  / solution dist=binary link=logit ;
 random intercept / subject = id ;
 by   _imputation_  ;
 nloptions tech=newrap ;
 ods output ParameterEstimates=outests ;
run;
proc mianalyze parms=outests;
 modeleffects sex ethnicity ;
run;

proc glimmix data=<10 datasets with missing values imputed> noclprint order=formatted;
 model frailty2 (event='1') = age age^2 sex ethnicity time mstatus
                                  / solution dist=binary link=logit ;
 random intercept / subject = id ;
 by   _imputation_  ;
```

```
 nloptions tech=newrap ;
ods output ParameterEstimates=outests ;
run;
proc mianalyze parms=outests;
 modeleffects mstatus ;
run;

proc glimmix data=<10 datasets with missing values imputed> noclprint order=formatted;
 model frailty2 (event='1') = age age^2 sex ethnicity time sesfactor
                                   / solution dist=binary link=logit ;
 random intercept / subject = id ;
 by   _imputation_  ;
 nloptions tech=newrap ;
 ods output ParameterEstimates=outests ;
run;
proc mianalyze parms=outests;
 modeleffects sesfactor ;
run;
```

Models for Tables 2 and 3

```
proc glimmix data=<10 datasets with missing values imputed> noclprint order=formatted;
 model frailty2 (event='1') = age age^2 sex ethnicity time riskfactor
                                   / solution dist=binary link=logit ;
 random intercept / subject = id ;
 by   _imputation_  ;
 nloptions tech=newrap ;
 ods output ParameterEstimates=outests ;
run;
proc mianalyze parms=outests;
 modeleffects riskfactor ;
run;
```

Models for Table 4

Base model (M0)

```
proc glimmix data=<10 datasets with missing values imputed> noclprint order=formatted;
model frailty2 (event='1') = age age^2 sex ethnicity mstatus mstatus*sex time ses
                          / solution dist=binary link=logit ;
 random intercept / subject = id ;
 by  _imputation_  ;
 nloptions tech=newrap ;
 ods output ParameterEstimates=outests ;  run;
proc mianalyze parms=outests;  modeleffects ses ;
run;
```

Base model + risk factor (M1)

proc glimmix data=<10 datasets with missing values imputed> noclprint order=formatted;
model frailty2 (event='1') = age age^{2} sex ethnicity mstatus mstatus*sex time riskfactor ses
/ solution dist=binary link=logit ;
random intercept / subject = id ;
by _imputation_ ;
nloptions tech=newrap ;
ods output ParameterEstimates=outests ; run;
proc mianalyze parms=outests; modeleffects ses ;
run;

Percentage change in ses = (ses from M1 – ses from M0)*100 / (ses from M0)

Models for Table 5

Base model (M0) – as for Table 4

Base model + set of risk factors as specified in Table 5 (M2)

proc glimmix data=<10 datasets with missing values imputed> noclprint order=formatted;
model frailty2 (event='1') = age age^{2} sex ethnicity mstatus mstatus*sex time <set of risk factors> ses
/ solution dist=binary link=logit ;
random intercept / subject = id ;
by _imputation_ ;
nloptions tech=newrap ;
ods output ParameterEstimates=outests ; run;
proc mianalyze parms=outests; modeleffects ses ;
run;

Percentage change in ses = (ses from M2 – ses from M0)*100 / (ses from M0)

Figure A1: Number and proportion of 6233 participants with 1, 2 and 3 frailty measurements by clinic

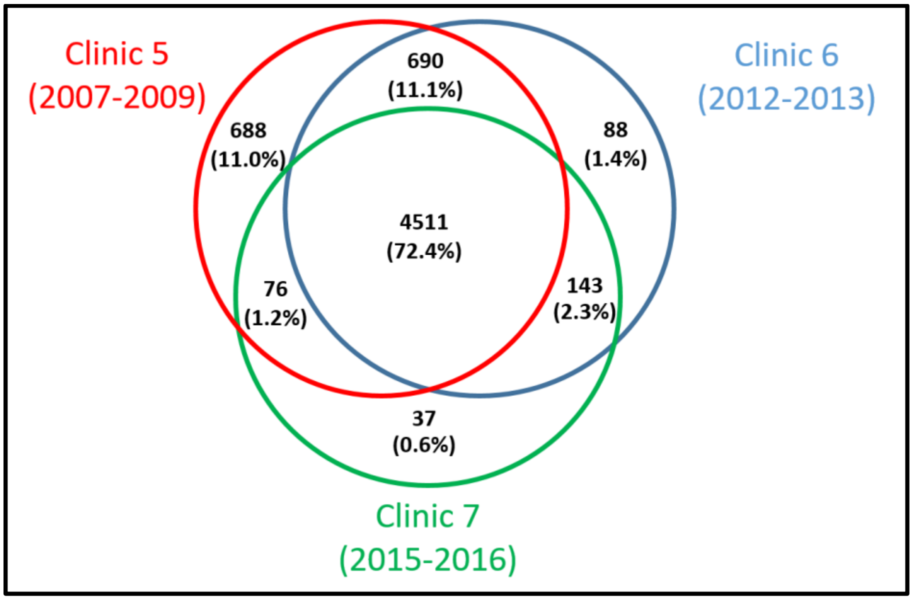

Table A1: Age standardised prevalence (%, and 95% confidence interval) of the components of frailty by employment grade among 16164 person-observations, by sex

		Employment grade			
Components of frailty	Sex	High	Intermediate	Low	p-trend
Physical activity	Men	22.6 (21.6, 23.7)	29.4 (28.2, 30.7)	47.1 (42.4, 51.8)	<0.001
	Women	28.5 (25.6, 32.5)	28.4 (26.5, 30.4)	39.1 (36.4, 41.8)	<0.001
Walking speed	Men	2.6 (2.2, 3.0)	4.9 (4.3, 5.5)	12.9 (9.8, 15.9)	<0.001
	Women	6.2 (4.5, 7.9)	8.8 (7.5, 10.0)	17.4 (15.4, 19.4)	<0.001
Grip strength	Men	13.3 (12.5, 14.2)	17.0 (15.9, 18.0)	28.3 (24.2, 32.3)	<0.001
	Women	13.9 (11.5, 14.2)	16.9 (15.2, 18.5)	21.6 (19.4, 23.8)	<0.001
Weight loss	Men	2.7 (2.3, 3.2)	2.8 (2.3, 3.3)	4.6 (2.6, 6.7)	0.19
	Women	4.8 (3.5, 6.2)	7.1 (6.0, 8.2)	6.8 (5.4, 8.2)	0.18
Exhaustion	Men	8.2 (7.5, 8.8)	10.3 (9.5, 11.2)	14.1 (10.8, 17.4)	<0.001
	Women	10.7 (8.7, 12.8)	14.5 (12.9, 16.0)	16.1 (14.0, 18.2)	<0.001

Table A2: Age standardised prevalence (%) by employment grade of the components of frailty among 562 person-observations with frailty, by sex

		Employment grade			
Components of frailty	Sex	High	Intermediate	Low	p-trend
Physical activity	Men	95.3	94.4	96.2	0.82
	Women	88.5	90.0	90.2	0.76
Walking speed	Men	57.1	62.2	67.1	0.21
	Women	51.7	68.3	83.6	<0.001
Grip strength	Men	90.5	83.4	88.6	0.27
	Women	83.6	71.4	76.6	0.82
Weight loss	Men	21.8	22.1	26.3	0.68
	Women	20.5	35.5	23.2	0.24
Exhaustion	Men	65.2	58.9	51.3	0.12
	Women	77.4	71.9	59.8	0.05

Table A3: Comparison of the prevalence of risk factors in participants in the analytic sample and those excluded, and their associations with employment grade measured at the start of the Whitehall II study (1986-1988).

Risk factor[a]	In analytic sample	N	Employment grade[a]: Total	High	Intermediate	Low	p-trend across grades
			Prevalence of risk factor (%[b])				
Current Smoker							
	Yes	6195	13.8	10.1	14.7	26.4	<0.001
	No[c]	3163	22.7	13.4	21.4	33.8	<0.001
Inactive							
	Yes	5953	12.9	8.5	11.3	30.8	<0.001
	No	2932	19.2	9.1	13.6	37.1	<0.001
Overweight or obese (BMI ≥ 25.0 Kgm^{-2})							
	Yes	6228	37.1	31.6	37.3	43.4	<0.001
	No	3187	41.7	37.8	39.3	47.0	<0.001
IL6 > 2.0 pg ml^{-1}							
	Yes	5080	39.3	33.7	41.0	48.7	<0.001
	No	510	51.6	39.8	49.5	65.9	<0.001

[a] Employment grade and the risk factors, smoking status, physical activity and obesity are from the start of the study (1986-1988) and IL6 is from the fourth clinic (2002-2004)

[b] Percentages are adjusted for age and sex

[c] Participants who were still alive at the fifth clinic (2007-2009) when frailty was first assessed but who are not in the analytic sample

Table A4: Effect of adjustment for each potential contributing factor on the association of frailty at 2007-2009, 2012-2013 or 2015-2016 with trend in last known employment grade at age 50, in men and women

Model adjustments	MEN (11691 person-observations)		WOMEN (4473 person-observations)	
	OR[a] (95% CI)	% Change[b]	OR[a] (95% CI)	% Change[b]
Base model[c]	1.36 (1.08,1.71)	Reference	1.65 (1.31, 2.09)	Reference
Base model + Smoking status	1.33 (1.06,1.68)	-5.8	1.63 (1.28, 2.06)	-3.2
Base model + Alcohol consumption	1.34 (1.06,1.69)	-4.7	1.54 (1.21, 1.97)	-13.4
Base model + Frequency of fruit/vegetable consumption	1.34 (1.06,1.68)	-5.0	1.64 (1.29, 2.08)	-1.4
Base model + Physical activity	1.27 (1.01,1.60)	-22.2	1.58 (1.24, 2.00)	-9.2
Base model + FEV	1.28 (1.01,1.61)	-19.8	1.61 (1.27, 2.05)	-4.7
Base model + BMI category	1.31 (1.04,1.65)	-11.9	1.58 (1.25, 2.01)	-8.8
Base model + Depressive symptoms	1.37 (1.08,1.72)	+2.0	1.69 (1.34, 2.15)	+5.0
Base model + Hypertension	1.38 (1.10,1.74)	-1.1	1.66 (1.31, 2.10)	+1.0
Base model + Diabetes	1.34 (1.06,1.68)	-4.3	1.65 (1.31, 2.09)	-0.1
Base model + CVD	1.35 (1.07,1.70)	-1.8	1.65 (1.30, 2.09)	-0.3
Base model + Total cholesterol	1.36 (1.08,1.71)	+0.7	1.65 (1.31, 2.09)	+0.1
Base model + HDL cholesterol	1.33 (1.06,1.68)	-6.5	1.61 (1.27, 2.04)	-5.6
Base model + Total cholesterol:HDL ratio	1.34 (1.07,1.69)	-3.9	1.62 (1.28, 2.05)	-4.2
Base model + Fasting glucose[d]	1.38 (1.09,1.75)	+1.1	1.67 (1.32, 2.12)	+1.6
Base model + IL6	1.29 (1.02,1.63)	-16.4	1.58 (1.24, 2.01)	-8.8
Base model + CRP	1.31 (1.04,1.65)	-12.0	1.58 (1.25, 2.01)	-8.7

[a] Odds ratios of frailty from trend with last known grade on 1 degree of freedom (i.e. the odds ratio of frailty for one unit lower grade level, across low, intermediate and high employment grades).

[b] Percentage change in coefficient (log odds ratio) for trend across employment grade, compared to base model.

[c] Base model is adjusted for age and age squared at fifth clinic, time of frailty measure since fifth clinic, sex, ethnicity, marital status, marital status by sex interaction.

[d] Fasting glucose in non-diabetics only

Table A5: Effect of adjustment for potential contributing factors on the association of frailty at 2007-2009, 2012-2013 or 2012-2013 with trend in last known employment grade at age 50, in men and women

		MEN (11691 person-observations)		WOMEN (4473 person-observations)	
Model	Model adjustments	OR[a] (95% CI)	% Change[b]	OR[a] (95% CI)	% Change[b]
Base model (BM)	Age at fifth clinic, age squared, sex, ethnicity, marital status, marital status by sex interaction, time of frailty measure since fifth clinic	1.36 (1.08, 1.71)	Reference	1.65 (1.31, 2.09)	Reference
Health behaviours	BM + smoking, alcohol, physical activity, fruit/vegetable consumption	1.24 (0.97, 1.57)	-30.8	1.47 (1.15, 1.89)	-23.3
Health behaviours + BMI	BM + smoking, alcohol, physical activity, fruit/vegetable consumption, BMI	1.21 (0.95, 1.53)	-38.4	1.43 (1.11, 1.84)	-28.8
Disease status	BM + prevalent CVD, diabetes and depressive symptoms	1.34 (1.06, 1.69)	-4.1	1.69 (1.33, 2.14)	+4.6
Predictors of impaired functioning:					
I: Of both cognitive and physical functioning	BM + hypertension, physical activity, FEV	1.21 (0.95, 1.53)	-38.5	1.55 (1.22, 1.97)	-12.9
II: As for I + physical function predictors	BM + hypertension, physical activity, FEV, inflammatory markers (CRP, IL6)	1.16 (0.92, 1.47)	-50.5	1.47 (1.15, 1.88)	-22.7
All covariates (AC)	BM + smoking, alcohol, physical activity, fruit/vegetable consumption, BMI, hypertension, physical activity, FEV, inflammatory markers, HDL, prevalent CVD, diabetes and depressive symptoms	1.13 (0.89, 1.45)	-58.6	1.44 (1.11, 1.87)	-27.3
SENSITIVITY ANALYSIS					
AC, excluding physical activity	As above, excluding physical activity	1.17 (0.92, 1.50)	-47.7	1.48 (1.14, 1.92)	-21.9
AC, excluding BMI	As above, excluding BMI	1.14 (0.90, 1.46)	-55.7	1.43 (1.11, 1.86)	-28.4
AC, excluding physical activity and BMI	As above, excluding physical activity and BMI	1.19 (0.93, 1.51)	-43.9	1.47 (1.14, 1.91)	-22.7

[a] Odds ratios of frailty from trend with last known grade on 1 degree of freedom (i.e. the odds ratio of frailty for one unit lower grade level, across low, intermediate and high employment grades), in 4456 men and 1777 women.

[b] Percentage change in coefficient (log odds ratio) for trend across employment grade, compared to base model.

[c] Base model is adjusted for age and age squared at fifth clinic, time of frailty measure since fifth clinic, sex, ethnicity, marital status, marital status by sex interaction.

Table A6: Effect of adjustment for each potential contributing factor on the association of ever having frailty at 2007-2009, 2012-2013 or 2015-2016 with last known employment grade at age 50 among 6233 participants[a]

Model adjustments	OR[b] (95% CI)	% Change[c] (95% CI)
Base model[d]	1.47 (1.25, 1.73)	Reference
Base model + Smoking status	1.44 (1.22, 1.69)	-6.1 (-15.4, -1.1)
Base model + Alcohol consumption	1.44 (1.22, 1.70)	-5.4 (-16.7, 3.0)
Base model + Frequency of fruit/vegetable consumption	1.45 (1.23, 1.71)	-3.9 (-11.2, 1.1)
Base model + Physical activity	1.40 (1.19, 1.65)	-12.6 (-25.7, -6.1)
Base model + FEV	1.42 (1.21, 1.67)	-9.6 (-19.3, -4.5)
Base model + BMI category	1.42 (1.21, 1.67)	-9.5 (-20.3, -3.9)
Base model + Depressive symptoms	1.50 (1.27, 1.76)	+4.0 (1.0, 10.3)
Base model + Hypertension	1.48 (1.26, 1.73)	+0.5 (-1.0, 3.4)
Base model + Diabetes	1.47 (1.25, 1.73)	-0.7 (-3.9, 0.2)
Base model + CVD	1.47 (1.25, 1.72)	-1.2 (-4.8, 0.1)
Base model + Total cholesterol	1.47 (1.25, 1.73)	0.0 (-1.0, 1.0)
Base model + HDL cholesterol	1.45 (1.23, 1.70)	-4.6 (-11.8, -1.2)
Base model + Total cholesterol:HDL ratio	1.45 (1.23, 1.70)	-4.0 (-9.8, -1.1)
Base model + Fasting glucose[e]	1.48 (1.25, 1.74)	+1.0 (-0.9, 4.3)
Base model + IL6	1.42 (1.20, 1.67)	-9.9 (-21.1, -4.7)
Base model + CRP	1.43 (1.21, 1.68)	-8.1 (-17.7, -3.5)

[a] Among the 6233 participants, 449 were classified as being frail on one or more occasions.

[b] Odds ratios of ever being frail at 2007-2009, 2012-2013 or 2015-2016 from trend with last known grade (ie the odds ratio of ever being frail associated with a change in one level of grade category (from high to intermediate or intermediate to low grade).

[c] Percentage change in coefficient (log odds ratio) for trend across employment grade, compared to base model

[d] Base model is adjusted for age at fifth clinic, sex, ethnicity, marital status, marital status by sex interaction

[e] Fasting glucose in non-diabetics only

Table S7: Effect of adjustment for potential contributing factors on the association of ever having frailty at 2007-2009, 2012-2013 or 2015-2016 with last known employment grade at age 50 among 6233 participants[a]

Model	Model adjustments	OR[b] (95% CI)	% Change[c] (95%CI)
Base model (BM)	Age at fifth clinic, sex, ethnicity, marital status, marital status by sex interaction	1.47 (1.25, 1.73)	Reference
Health behaviours	BM + smoking, alcohol, physical activity, fruit/vegetable consumption	1.35 (1.14, 1.59)	-22.8 (-47.5, -9.5)
Health behaviours + BMI	BM + smoking, alcohol, physical activity, fruit/vegetable consumption, BMI	1.31 (1.11, 1.56)	-29.5 (-59.3, -14.4)
Disease status	BM + prevalent CVD, diabetes and depressive symptoms	1.48 (1.26, 1.75)	+2.1 (-2.4, 7.7)
Predictors of impaired functioning:			
I: Of both cognitive and physical functioning	BM + hypertension, physical activity, FEV	1.36 (1.15, 1.60)	-20.7 (-40.0, -11.2)
II: As for I + physical function predictors	BM + hypertension, physical activity, FEV, inflammatory markers (CRP, IL6)	1.31 (1.11, 1.55)	-30.1 (-56.7, -18.0)
All covariates (AC)	BM + smoking, alcohol, physical activity, fruit/vegetable consumption, BMI, hypertension, physical activity, FEV, inflammatory markers, HDL, prevalent CVD, diabetes and depressive symptoms	1.28 (1.08, 1.52)	-35.8 (-70.6, -17.5)
SENSITIVITY ANALYSIS			
AC, excluding physical activity	As above, excluding physical activity	1.31 (1.11, 1.56)	-29.5 (-60.5, -13.5)
AC, excluding BMI	As above, excluding BMI	1.29 (1.09, 1.54)	-33.7 (-66.7, -16.8)
AC, excluding physical activity and BMI	As above, excluding physical activity and BMI	1.33 (1.12, 1.58)	-26.9 (-56.1, -11.3)

[a] Among the 6233 participants, 449 were classified as being frail on one or more occasions.

[b] Odds ratios of frailty from trend with last known grade (i.e. the odds ratio of frailty for one category lower employment grade (from high to intermediate or intermediate to low employment grade), in 6233 participants.

[c] Percentage change in coefficient (log odds ratio) for trend across employment grade, compared to base model

Table A8: Frailty and mortality outcomes and attrition by employment grade among 6233 participants

			Employment grade		
Outcome	Explanation	Total (N=6233)	High (N=2677)	Intermediate (N=2764)	Low (N=792)
Frail	Classified as being frail on at least one occasion at Clinic 4, 5 or 6	449	116 (4.3%)	200 (7.2%)	133 (16.8%)
Dead	Not frail but died before assessment at clinic 6	282	111 (4.2%)	134 (4.9%)	37 (4.8%)
Attrition	Not frail but lost to follow-up before assessment at clinic 6	1035	329 (12.3%)	484 (17.5%)	222 (28.0%)
Followed -up	Not frail at (or before) clinic 6	4467	2121 (79.3%)	1946 (70.4%)	400 (50.5%)
